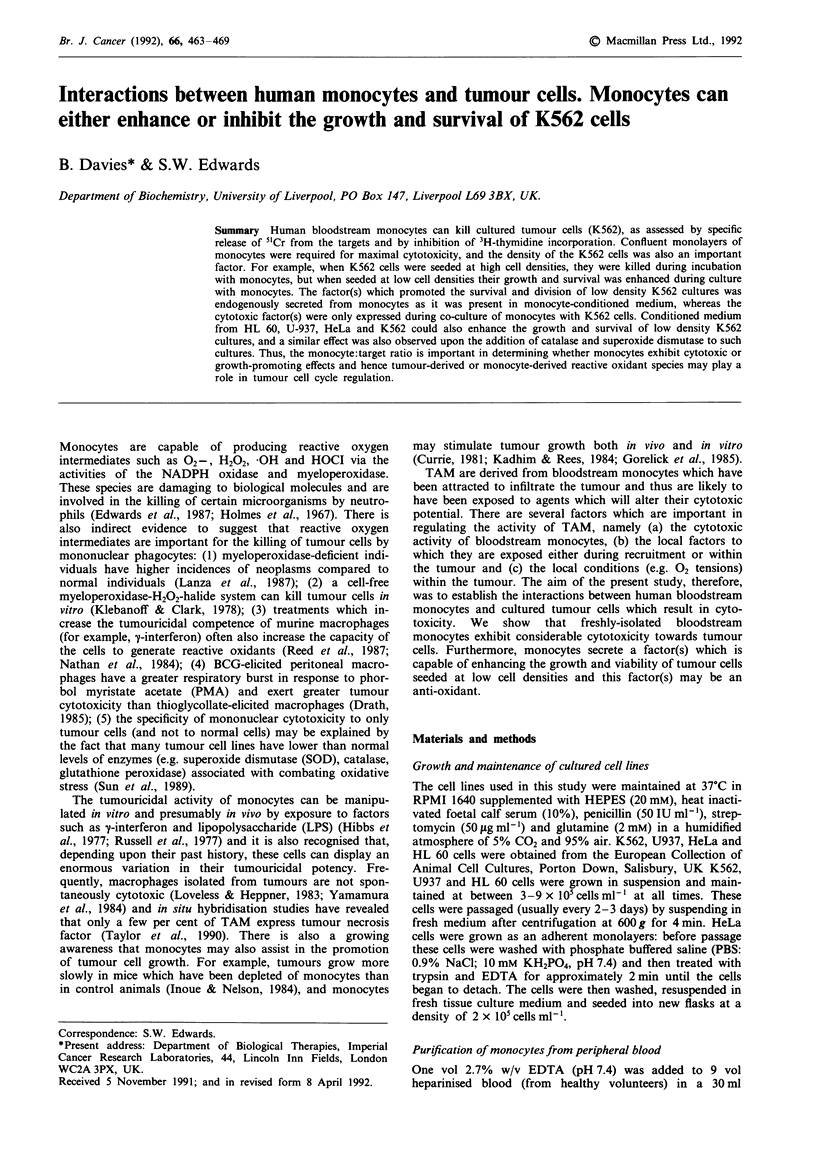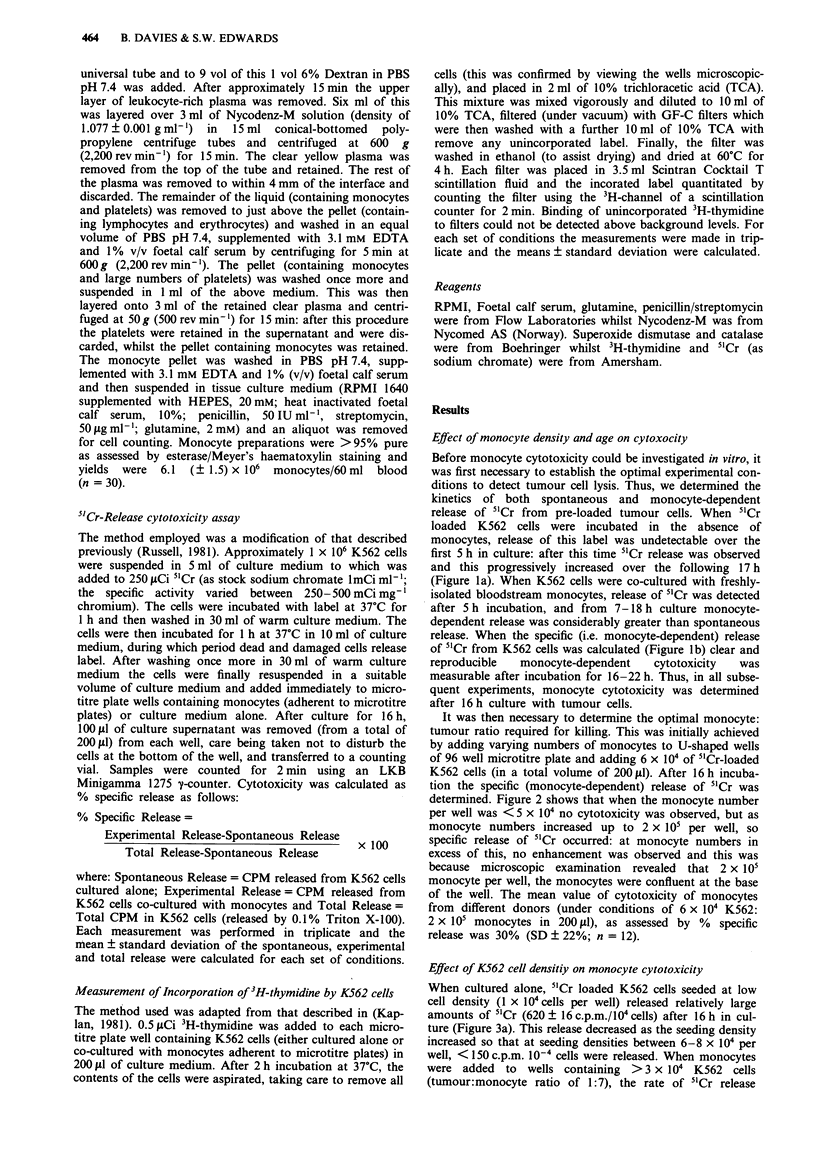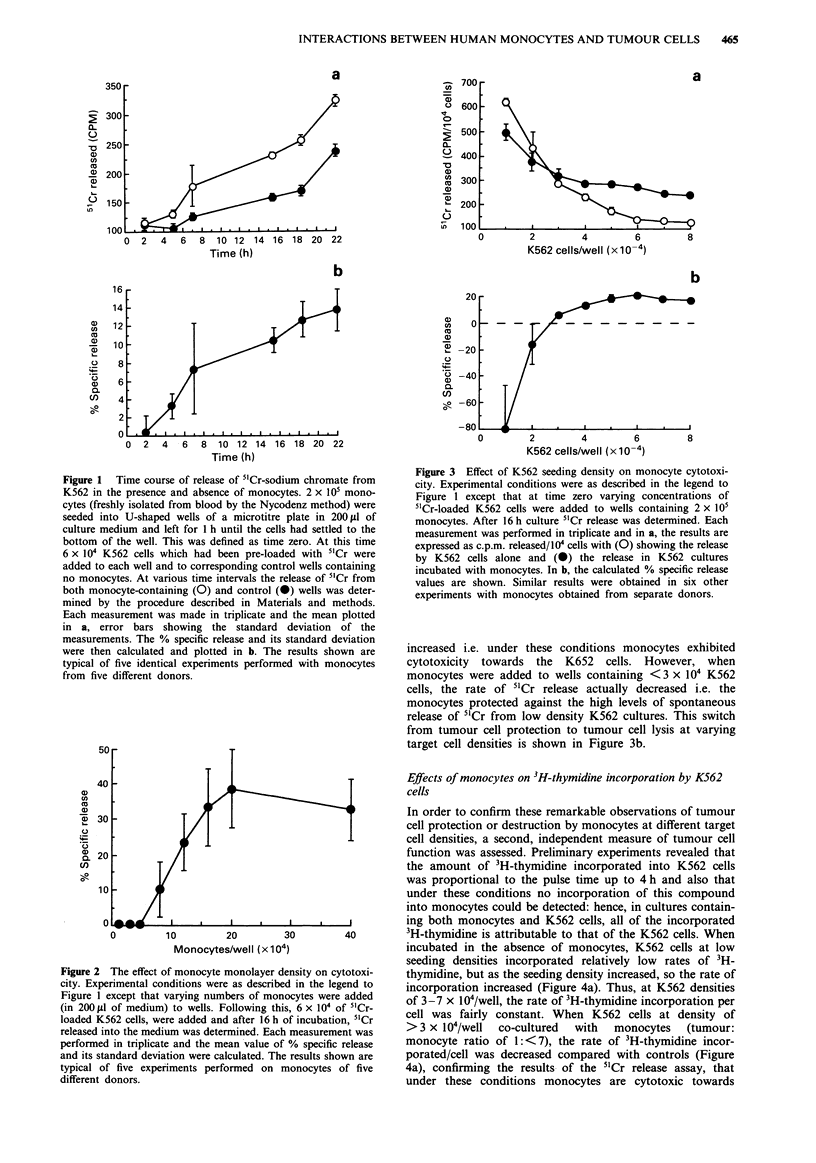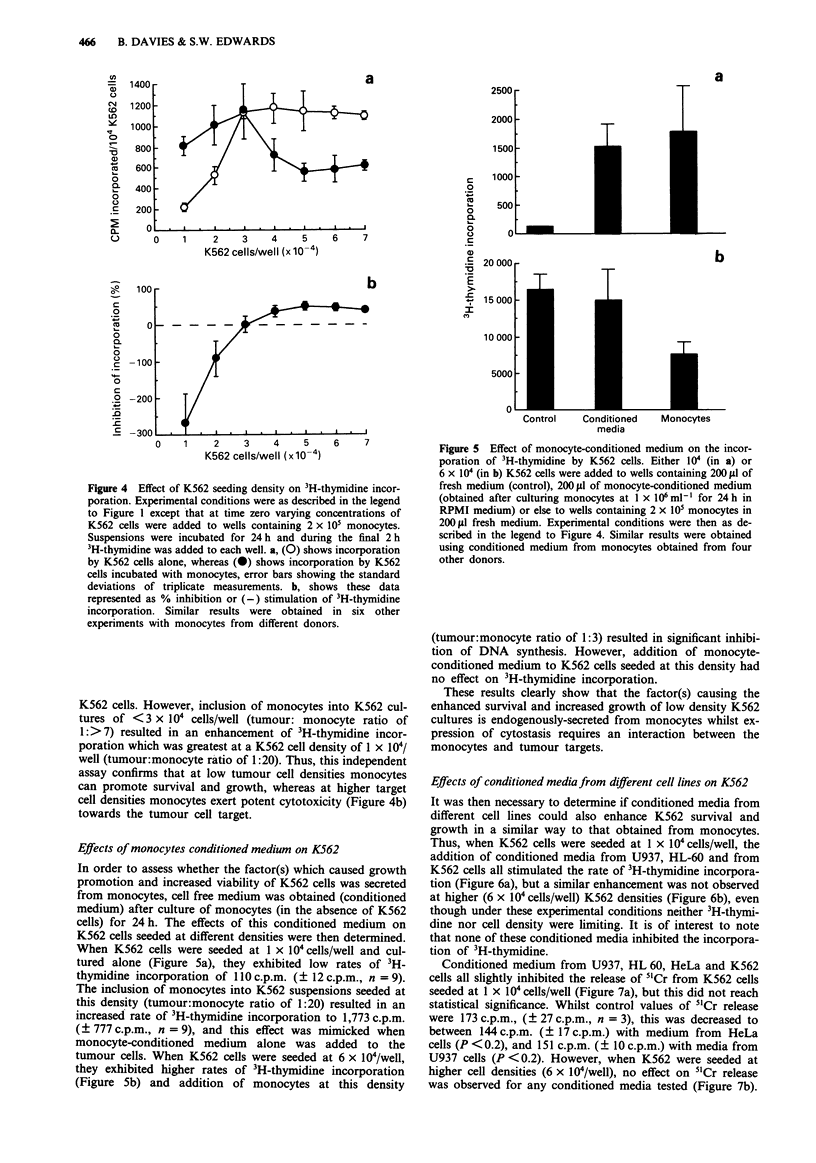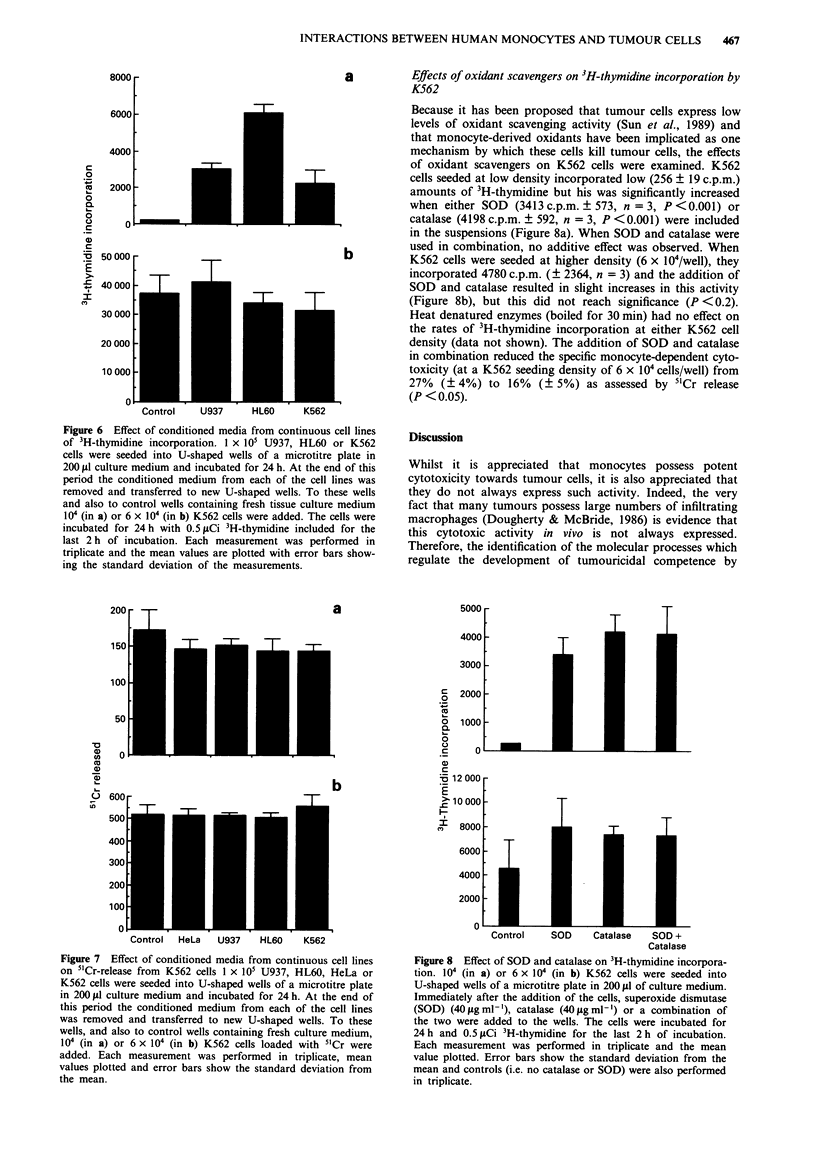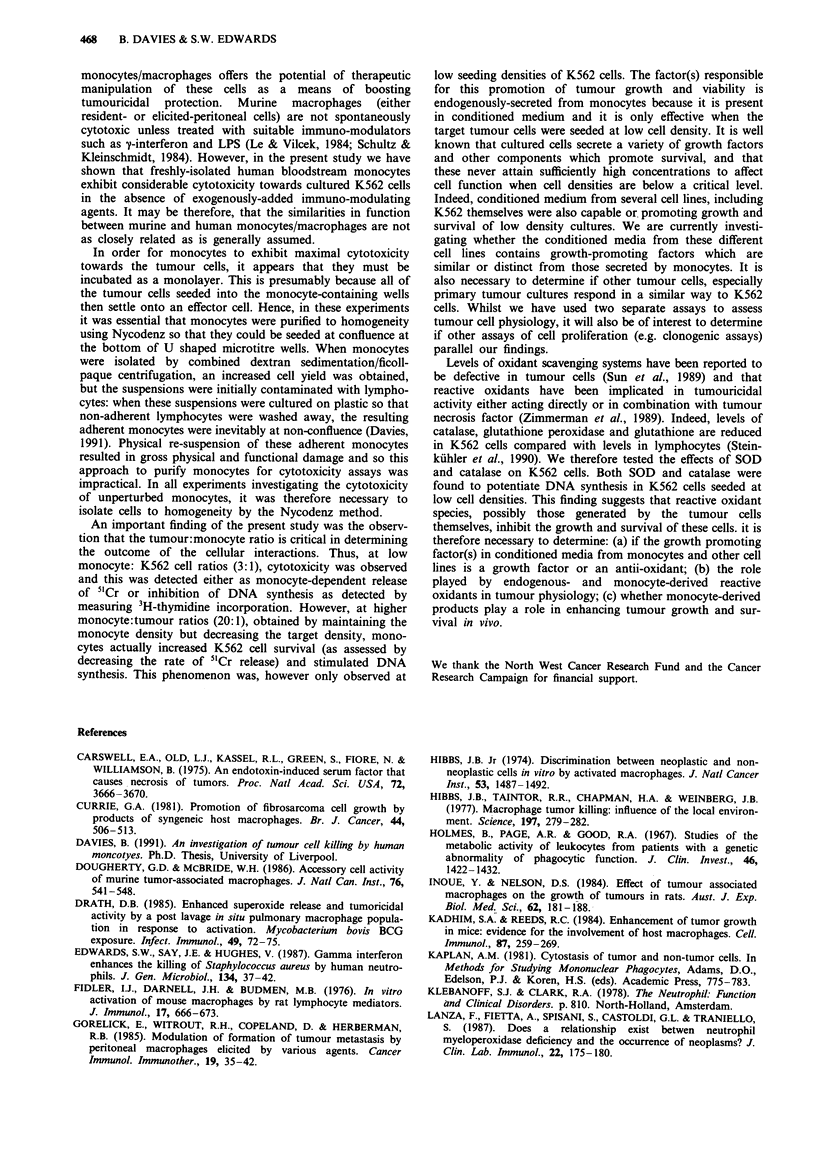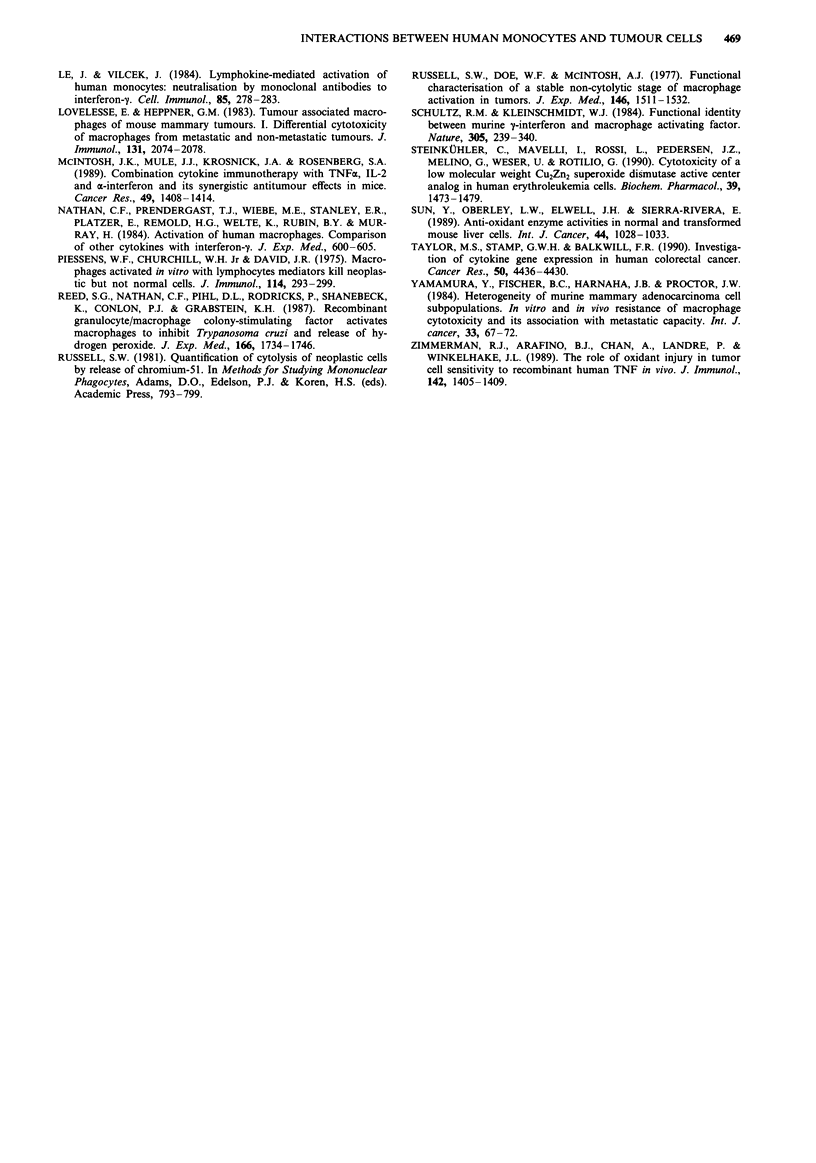# Interactions between human monocytes and tumour cells. Monocytes can either enhance or inhibit the growth and survival of K562 cells.

**DOI:** 10.1038/bjc.1992.296

**Published:** 1992-09

**Authors:** B. Davies, S. W. Edwards

**Affiliations:** Department of Biochemistry, University of Liverpool, UK.

## Abstract

Human bloodstream monocytes can kill cultured tumour cells (K562), as assessed by specific release of 51Cr from the targets and by inhibition of 3H-thymidine incorporation. Confluent monolayers of monocytes were required for maximal cytotoxicity, and the density of the K562 cells was also an important factor. For example, when K562 cells were seeded at high cell densities, they were killed during incubation with monocytes, but when seeded at low cell densities their growth and survival was enhanced during culture with monocytes. The factor(s) which promoted the survival and division of low density K562 cultures was endogenously secreted from monocytes as it was present in monocyte-conditioned medium, whereas the cytotoxic factor(s) were only expressed during co-culture of monocytes with K562 cells. Conditioned medium from HL 60, U-937, HeLa and K562 could also enhance the growth and survival of low density K562 cultures, and a similar effect was also observed upon the addition of catalase and superoxide dismutase to such cultures. Thus, the monocyte:target ratio is important in determining whether monocytes exhibit cytotoxic or growth-promoting effects and hence tumour-derived or monocyte-derived reactive oxidant species may play a role in tumour cell cycle regulation.


Br. J. Cancer (1992), 66, 463-469 © Macmillan Press Ltd., 1992

### Interactions between human monocytes and tumour cells. Monocytes can either enhance or inhibit the growth and survival of K562 cells

B. Davies* & S.W. Edwards

*Department of Biochemistry, University of Liverpool, PO Box 147, Liverpool L69 3BX, UK.*

**Summary** Human bloodstream monocytes can kill cultured tumour cells (K562), as assessed by specific release of $^{51}Cr$ from the targets and by inhibition of $^{3}H$-thymidine incorporation. Confluent monolayers of monocytes were required for maximal cytotoxicity, and the density of the K562 cells was also an important factor. For example, when K562 cells were seeded at high cell densities, they were killed during incubation with monocytes, but when seeded at low cell densities their growth and survival was enhanced during culture with monocytes. The factor(s) which promoted the survival and division of low density K562 cultures was endogenously secreted from monocytes as it was present in monocyte-conditioned medium, whereas the cytotoxic factor(s) were only expressed during co-culture of monocytes with K562 cells. Conditioned medium from HL 60, U-937, HeLa and K562 could also enhance the growth and survival of low density K562 cultures, and a similar effect was also observed upon the addition of catalase and superoxide dismutase to such cultures. Thus, the monocyte:target ratio is important in determining whether monocytes exhibit cytotoxic or growth-promoting effects and hence tumour-derived or monocyte-derived reactive oxidant species may play a role in tumour cell cycle regulation.

Monocytes are capable of producing reactive oxygen intermediates such as $O_2-$, $H_2O_2$, ·OH and HOCl via the activities of the NADPH oxidase and myeloperoxidase. These species are damaging to biological molecules and are involved in the killing of certain microorganisms by neutrophils (Edwards *et al.*, 1987; Holmes *et al.*, 1967). There is also indirect evidence to suggest that reactive oxygen intermediates are important for the killing of tumour cells by mononuclear phagocytes: (1) myeloperoxidase-deficient individuals have higher incidences of neoplasms compared to normal individuals (Lanza *et al.*, 1987); (2) a cell-free myeloperoxidase-$H_2O_2$-halide system can kill tumour cells *in vitro* (Klebanoff & Clark, 1978); (3) treatments which increase the tumouricidal competence of murine macrophages (for example, γ-interferon) often also increase the capacity of the cells to generate reactive oxidants (Reed *et al.*, 1987; Nathan *et al.*, 1984); (4) BCG-elicited peritoneal macrophages have a greater respiratory burst in response to phorbol myristate acetate (PMA) and exert greater tumour cytotoxicity than thioglycollate-elicited macrophages (Drath, 1985); (5) the specificity of mononuclear cytotoxicity to only tumour cells (and not to normal cells) may be explained by the fact that many tumour cell lines have lower than normal levels of enzymes (e.g. superoxide dismutase (SOD), catalase, glutathione peroxidase) associated with combating oxidative stress (Sun *et al.*, 1989).

The tumouricidal activity of monocytes can be manipulated *in vitro* and presumably *in vivo* by exposure to factors such as γ-interferon and lipopolysaccharide (LPS) (Hibbs *et al.*, 1977; Russell *et al.*, 1977) and it is also recognised that, depending upon their past history, these cells can display an enormous variation in their tumouricidal potency. Frequently, macrophages isolated from tumours are not spontaneously cytotoxic (Loveless & Heppner, 1983; Yamamura *et al.*, 1984) and *in situ* hybridisation studies have revealed that only a few per cent of TAM express tumour necrosis factor (Taylor *et al.*, 1990). There is also a growing awareness that monocytes may also assist in the promotion of tumour cell growth. For example, tumours grow more slowly in mice which have been depleted of monocytes than in control animals (Inoue & Nelson, 1984), and monocytes may stimulate tumour growth both *in vivo* and *in vitro* (Currie, 1981; Kadhim & Rees, 1984; Gorelick *et al.*, 1985).

TAM are derived from bloodstream monocytes which have been attracted to infiltrate the tumour and thus are likely to have been exposed to agents which will alter their cytotoxic potential. There are several factors which are important in regulating the activity of TAM, namely (a) the cytotoxic activity of bloodstream monocytes, (b) the local factors to which they are exposed either during recruitment or within the tumour and (c) the local conditions (e.g. $O_2$ tensions) within the tumour. The aim of the present study, therefore, was to establish the interactions between human bloodstream monocytes and cultured tumour cells which result in cytotoxicity. We show that freshly-isolated bloodstream monocytes exhibit considerable cytotoxicity towards tumour cells. Furthermore, monocytes secrete a factor(s) which is capable of enhancing the growth and viability of tumour cells seeded at low cell densities and this factor(s) may be an anti-oxidant.

### Materials and methods

### *Growth and maintenance of cultured cell lines*

The cell lines used in this study were maintained at 37°C in RPMI 1640 supplemented with HEPES (20 mM), heat inactivated foetal calf serum (10%), penicillin (50 IU $ml^{-1}$), streptomycin (50 $\mu g\ ml^{-1}$) and glutamine (2 mM) in a humidified atmosphere of 5% $CO_2$ and 95% air. K562, U937, HeLa and HL 60 cells were obtained from the European Collection of Animal Cell Cultures, Porton Down, Salisbury, UK K562, U937 and HL 60 cells were grown in suspension and maintained at between $3-9 \times 10^5$ cells $ml^{-1}$ at all times. These cells were passaged (usually every 2-3 days) by suspending in fresh medium after centrifugation at 600 *g* for 4 min. HeLa cells were grown as an adherent monolayers: before passage these cells were washed with phosphate buffered saline (PBS: 0.9% NaCl; 10 mM $KH_2PO_4$, pH 7.4) and then treated with trypsin and EDTA for approximately 2 min until the cells began to detach. The cells were then washed, resuspended in fresh tissue culture medium and seeded into new flasks at a density of $2 \times 10^5$ cells $ml^{-1}$.

### *Purification of monocytes from peripheral blood*

One vol 2.7% w/v EDTA (pH 7.4) was added to 9 vol heparinised blood (from healthy volunteers) in a 30 ml

Correspondence: S.W. Edwards.
*Present address: Department of Biological Therapies, Imperial Cancer Research Laboratories, 44, Lincoln Inn Fields, London WC2A 3PX, UK.
Received 5 November 1991; and in revised form 8 April 1992.

universal tube and to 9 vol of this 1 vol 6% Dextran in PBS pH 7.4 was added. After approximately 15 min the upper layer of leukocyte-rich plasma was removed. Six ml of this was layered over 3 ml of Nycodenz-M solution (density of 1.077 ± 0.001 g ml$^{-1}$) in 15 ml conical-bottomed polypropylene centrifuge tubes and centrifuged at 600 *g* (2,200 rev min$^{-1}$) for 15 min. The clear yellow plasma was removed from the top of the tube and retained. The rest of the plasma was removed to within 4 mm of the interface and discarded. The remainder of the liquid (containing monocytes and platelets) was removed to just above the pellet (containing lymphocytes and erythrocytes) and washed in an equal volume of PBS pH 7.4, supplemented with 3.1 mM EDTA and 1% v/v foetal calf serum by centrifuging for 5 min at 600 *g* (2,200 rev min$^{-1}$). The pellet (containing monocytes and large numbers of platelets) was washed once more and suspended in 1 ml of the above medium. This was then layered onto 3 ml of the retained clear plasma and centrifuged at 50 *g* (500 rev min$^{-1}$) for 15 min: after this procedure the platelets were retained in the supernatant and were discarded, whilst the pellet containing monocytes was retained. The monocyte pellet was washed in PBS pH 7.4, supplemented with 3.1 mM EDTA and 1% (v/v) foetal calf serum and then suspended in tissue culture medium (RPMI 1640 supplemented with HEPES, 20 mM; heat inactivated foetal calf serum, 10%; penicillin, 50 IU ml$^{-1}$, streptomycin, 50 μg ml$^{-1}$; glutamine, 2 mM) and an aliquot was removed for cell counting. Monocyte preparations were >95% pure as assessed by esterase/Meyer's haematoxylin staining and yields were 6.1 (± 1.5) × 10$^6$ monocytes/60 ml blood (*n* = 30).

*$^{51}$Cr-Release cytotoxicity assay*

The method employed was a modification of that described previously (Russell, 1981). Approximately 1 × 10$^6$ K562 cells were suspended in 5 ml of culture medium to which was added to 250 μCi $^{51}$Cr (as stock sodium chromate 1mCi ml$^{-1}$; the specific activity varied between 250–500 mCi mg$^{-1}$ chromium). The cells were incubated with label at 37°C for 1 h and then washed in 30 ml of warm culture medium. The cells were then incubated for 1 h at 37°C in 10 ml of culture medium, during which period dead and damaged cells release label. After washing once more in 30 ml of warm culture medium the cells were finally resuspended in a suitable volume of culture medium and added immediately to microtitre plate wells containing monocytes (adherent to microtitre plates) or culture medium alone. After culture for 16 h, 100 μl of culture supernatant was removed (from a total of 200 μl) from each well, care being taken not to disturb the cells at the bottom of the well, and transferred to a counting vial. Samples were counted for 2 min using an LKB Minigamma 1275 γ-counter. Cytotoxicity was calculated as % specific release as follows:

$$\% \text{ Specific Release} = \frac{\text{Experimental Release-Spontaneous Release}}{\text{Total Release-Spontaneous Release}} \times 100$$

where: Spontaneous Release = CPM released from K562 cells cultured alone; Experimental Release = CPM released from K562 cells co-cultured with monocytes and Total Release = Total CPM in K562 cells (released by 0.1% Triton X-100). Each measurement was performed in triplicate and the mean ± standard deviation of the spontaneous, experimental and total release were calculated for each set of conditions.

*Measurement of Incorporation of $^3$H-thymidine by K562 cells*

The method used was adapted from that described in (Kaplan, 1981). 0.5 μCi $^3$H-thymidine was added to each microtitre plate well containing K562 cells (either cultured alone or co-cultured with monocytes adherent to microtitre plates) in 200 μl of culture medium. After 2 h incubation at 37°C, the contents of the cells were aspirated, taking care to remove all cells (this was confirmed by viewing the wells microscopically), and placed in 2 ml of 10% trichloracetic acid (TCA). This mixture was mixed vigorously and diluted to 10 ml of 10% TCA, filtered (under vacuum) with GF-C filters which were then washed with a further 10 ml of 10% TCA with remove any unincorporated label. Finally, the filter was washed in ethanol (to assist drying) and dried at 60°C for 4 h. Each filter was placed in 3.5 ml Scintran Cocktail T scintillation fluid and the incorated label quantitated by counting the filter using the $^3$H-channel of a scintillation counter for 2 min. Binding of unincorporated $^3$H-thymidine to filters could not be detected above background levels. For each set of conditions the measurements were made in triplicate and the means ± standard deviation were calculated.

*Reagents*

RPMI, Foetal calf serum, glutamine, penicillin/streptomycin were from Flow Laboratories whilst Nycodenz-M was from Nycomed AS (Norway). Superoxide dismutase and catalase were from Boehringer whilst $^3$H-thymidine and $^{51}$Cr (as sodium chromate) were from Amersham.

### Results

*Effect of monocyte density and age on cytoxocity*

Before monocyte cytotoxicity could be investigated *in vitro*, it was first necessary to establish the optimal experimental conditions to detect tumour cell lysis. Thus, we determined the kinetics of both spontaneous and monocyte-dependent release of $^{51}$Cr from pre-loaded tumour cells. When $^{51}$Cr loaded K562 cells were incubated in the absence of monocytes, release of this label was undetectable over the first 5 h in culture: after this time $^{51}$Cr release was observed and this progressively increased over the following 17 h (Figure 1a). When K562 cells were co-cultured with freshly-isolated bloodstream monocytes, release of $^{51}$Cr was detected after 5 h incubation, and from 7–18 h culture monocyte-dependent release was considerably greater than spontaneous release. When the specific (i.e. monocyte-dependent) release of $^{51}$Cr from K562 cells was calculated (Figure 1b) clear and reproducible monocyte-dependent cytotoxicity was measurable after incubation for 16–22 h. Thus, in all subsequent experiments, monocyte cytotoxicity was determined after 16 h culture with tumour cells.

It was then necessary to determine the optimal monocyte: tumour ratio required for killing. This was initially achieved by adding varying numbers of monocytes to U-shaped wells of 96 well microtitre plate and adding 6 × 10$^4$ of $^{51}$Cr-loaded K562 cells (in a total volume of 200 μl). After 16 h incubation the specific (monocyte-dependent) release of $^{51}$Cr was determined. Figure 2 shows that when the monocyte number per well was <5 × 10$^4$ no cytotoxicity was observed, but as monocyte numbers increased up to 2 × 10$^5$ per well, so specific release of $^{51}$Cr occurred: at monocyte numbers in excess of this, no enhancement was observed and this was because microscopic examination revealed that 2 × 10$^5$ monocyte per well, the monocytes were confluent at the base of the well. The mean value of cytotoxicity of monocytes from different donors (under conditions of 6 × 10$^4$ K562: 2 × 10$^5$ monocytes in 200 μl), as assessed by % specific release was 30% (SD ± 22%; *n* = 12).

*Effect of K562 cell densitiy on monocyte cytotoxicity*

When cultured alone, $^{51}$Cr loaded K562 cells seeded at low cell density (1 × 10$^4$ cells per well) released relatively large amounts of $^{51}$Cr (620 ± 16 c.p.m./10$^4$ cells) after 16 h in culture (Figure 3a). This release decreased as the seeding density increased so that at seeding densities between 6–8 × 10$^4$ per well, <150 c.p.m. 10$^{-4}$ cells were released. When monocytes were added to wells containing >3 × 10$^4$ K562 cells (tumour:monocyte ratio of 1:7), the rate of $^{51}$Cr release

**Figure 1** Time course of release of $^{51}$Cr-sodium chromate from K562 in the presence and absence of monocytes. $2 \times 10^5$ monocytes (freshly isolated from blood by the Nycodenz method) were seeded into U-shaped wells of a microtitre plate in 200 μl of culture medium and left for 1 h until the cells had settled to the bottom of the well. This was defined as time zero. At this time $6 \times 10^4$ K562 cells which had been pre-loaded with $^{51}$Cr were added to each well and to corresponding control wells containing no monocytes. At various time intervals the release of $^{51}$Cr from both monocyte-containing (○) and control (●) wells was determined by the procedure described in Materials and methods. Each measurement was made in triplicate and the mean plotted in **a**, error bars showing the standard deviation of the measurements. The % specific release and its standard deviation were then calculated and plotted in **b**. The results shown are typical of five identical experiments performed with monocytes from five different donors.

**Figure 2** The effect of monocyte monolayer density on cytotoxicity. Experimental conditions were as described in the legend to Figure 1 except that varying numbers of monocytes were added (in 200 μl of medium) to wells. Following this, $6 \times 10^4$ of $^{51}$Cr-loaded K562 cells, were added and after 16 h of incubation, $^{51}$Cr released into the medium was determined. Each measurement was performed in triplicate and the mean value of % specific release and its standard deviation were calculated. The results shown are typical of five experiments performed on monocytes of five different donors.

**Figure 3** Effect of K562 seeding density on monocyte cytotoxicity. Experimental conditions were as described in the legend to Figure 1 except that at time zero varying concentrations of $^{51}$Cr-loaded K562 cells were added to wells containing $2 \times 10^5$ monocytes. After 16 h culture $^{51}$Cr release was determined. Each measurement was performed in triplicate and in **a**, the results are expressed as c.p.m. released/10$^4$ cells with (○) showing the release by K562 cells alone and (●) the release in K562 cultures incubated with monocytes. In **b**, the calculated % specific release values are shown. Similar results were obtained in six other experiments with monocytes obtained from separate donors.

increased i.e. under these conditions monocytes exhibited cytotoxicity towards the K652 cells. However, when monocytes were added to wells containing $<3 \times 10^4$ K562 cells, the rate of $^{51}$Cr release actually decreased i.e. the monocytes protected against the high levels of spontaneous release of $^{51}$Cr from low density K562 cultures. This switch from tumour cell protection to tumour cell lysis at varying target cell densities is shown in Figure 3b.

### *Effects of monocytes on $^3$H-thymidine incorporation by K562 cells*

In order to confirm these remarkable observations of tumour cell protection or destruction by monocytes at different target cell densities, a second, independent measure of tumour cell function was assessed. Preliminary experiments revealed that the amount of $^3$H-thymidine incorporated into K562 cells was proportional to the pulse time up to 4 h and also that under these conditions no incorporation of this compound into monocytes could be detected: hence, in cultures containing both monocytes and K562 cells, all of the incorporated $^3$H-thymidine is attributable to that of the K562 cells. When incubated in the absence of monocytes, K562 cells at low seeding densities incorporated relatively low rates of $^3$H-thymidine, but as the seeding density increased, so the rate of incorporation increased (Figure 4a). Thus, at K562 densities of 3–7 × 10$^4$/well, the rate of $^3$H-thymidine incorporation per cell was fairly constant. When K562 cells at density of $>3 \times 10^4$/well co-cultured with monocytes (tumour: monocyte ratio of 1:<7), the rate of $^3$H-thymidine incorporated/cell was decreased compared with controls (Figure 4a), confirming the results of the $^{51}$Cr release assay, that under these conditions monocytes are cytotoxic towards

**Figure 4** Effect of K562 seeding density on $^3$H-thymidine incorporation. Experimental conditions were as described in the legend to Figure 1 except that at time zero varying concentrations of K562 cells were added to wells containing $2 \times 10^5$ monocytes. Suspensions were incubated for 24 h and during the final 2 h $^3$H-thymidine was added to each well. **a**, (○) shows incorporation by K562 cells alone, whereas (●) shows incorporation by K562 cells incubated with monocytes, error bars showing the standard deviations of triplicate measurements. **b**, shows these data represented as % inhibition or (−) stimulation of $^3$H-thymidine incorporation. Similar results were obtained in six other experiments with monocytes from different donors.

**Figure 5** Effect of monocyte-conditioned medium on the incorporation of $^3$H-thymidine by K562 cells. Either $10^4$ (in **a**) or $6 \times 10^4$ (in **b**) K562 cells were added to wells containing 200 µl of fresh medium (control), 200 µl of monocyte-conditioned medium (obtained after culturing monocytes at $1 \times 10^6$ ml$^{-1}$ for 24 h in RPMI medium) or else to wells containing $2 \times 10^5$ monocytes in 200 µl fresh medium. Experimental conditions were then as described in the legend to Figure 4. Similar results were obtained using conditioned medium from monocytes obtained from four other donors.

K562 cells. However, inclusion of monocytes into K562 cultures of $< 3 \times 10^4$ cells/well (tumour: monocyte ratio of 1:>7) resulted in an enhancement of $^3$H-thymidine incorporation which was greatest at a K562 cell density of $1 \times 10^4$/well (tumour:monocyte ratio of 1:20). Thus, this independent assay confirms that at low tumour cell densities monocytes can promote survival and growth, whereas at higher target cell densities monocytes exert potent cytotoxicity (Figure 4b) towards the tumour cell target.

*Effects of monocytes conditioned medium on K562*

In order to assess whether the factor(s) which caused growth promotion and increased viability of K562 cells was secreted from monocytes, cell free medium was obtained (conditioned medium) after culture of monocytes (in the absence of K562 cells) for 24 h. The effects of this conditioned medium on K562 cells seeded at different densities were then determined. When K562 cells were seeded at $1 \times 10^4$ cells/well and cultured alone (Figure 5a), they exhibited low rates of $^3$H-thymidine incorporation of 110 c.p.m. (± 12 c.p.m., $n = 9$). The inclusion of monocytes into K562 suspensions seeded at this density (tumour:monocyte ratio of 1:20) resulted in an increased rate of $^3$H-thymidine incorporation to 1,773 c.p.m. (± 777 c.p.m., $n = 9$), and this effect was mimicked when monocyte-conditioned medium alone was added to the tumour cells. When K562 cells were seeded at $6 \times 10^4$/well, they exhibited higher rates of $^3$H-thymidine incorporation (Figure 5b) and addition of monocytes at this density (tumour:monocyte ratio of 1:3) resulted in significant inhibition of DNA synthesis. However, addition of monocyte-conditioned medium to K562 cells seeded at this density had no effect on $^3$H-thymidine incorporation.

These results clearly show that the factor(s) causing the enhanced survival and increased growth of low density K562 cultures is endogenously-secreted from monocytes whilst expression of cytostasis requires an interaction between the monocytes and tumour targets.

*Effects of conditioned media from different cell lines on K562*

It was then necessary to determine if conditioned media from different cell lines could also enhance K562 survival and growth in a similar way to that obtained from monocytes. Thus, when K562 cells were seeded at $1 \times 10^4$ cells/well, the addition of conditioned media from U937, HL-60 and from K562 cells all stimulated the rate of $^3$H-thymidine incorporation (Figure 6a), but a similar enhancement was not observed at higher ($6 \times 10^4$ cells/well) K562 densities (Figure 6b), even though under these experimental conditions neither $^3$H-thymidine nor cell density were limiting. It is of interest to note that none of these conditioned media inhibited the incorporation of $^3$H-thymidine.

Conditioned medium from U937, HL 60, HeLa and K562 cells all slightly inhibited the release of $^{51}$Cr from K562 cells seeded at $1 \times 10^4$ cells/well (Figure 7a), but this did not reach statistical significance. Whilst control values of $^{51}$Cr release were 173 c.p.m., (± 27 c.p.m., $n = 3$), this was decreased to between 144 c.p.m. (± 17 c.p.m.) with medium from HeLa cells ($P < 0.2$), and 151 c.p.m. (± 10 c.p.m.) with media from U937 cells ($P < 0.2$). However, when K562 were seeded at higher cell densities ($6 \times 10^4$/well), no effect on $^{51}$Cr release was observed for any conditioned media tested (Figure 7b).

**Figure 6** Effect of conditioned media from continuous cell lines of $^3$H-thymidine incorporation. $1 \times 10^5$ U937, HL60 or K562 cells were seeded into U-shaped wells of a microtitre plate in 200 µl culture medium and incubated for 24 h. At the end of this period the conditioned medium from each of the cell lines was removed and transferred to new U-shaped wells. To these wells and also to control wells containing fresh tissue culture medium $10^4$ (in **a**) or $6 \times 10^4$ (in **b**) K562 cells were added. The cells were incubated for 24 h with 0.5 µCi $^3$H-thymidine included for the last 2 h of incubation. Each measurement was performed in triplicate and the mean values are plotted with error bars showing the standard deviation of the measurements.

*Effects of oxidant scavengers on $^3$H-thymidine incorporation by K562*

Because it has been proposed that tumour cells express low levels of oxidant scavenging activity (Sun *et al.*, 1989) and that monocyte-derived oxidants have been implicated as one mechanism by which these cells kill tumour cells, the effects of oxidant scavengers on K562 cells were examined. K562 cells seeded at low density incorporated low (256 ± 19 c.p.m.) amounts of $^3$H-thymidine but his was significantly increased when either SOD (3413 c.p.m. ± 573, $n = 3$, $P < 0.001$) or catalase (4198 c.p.m. ± 592, $n = 3$, $P < 0.001$) were included in the suspensions (Figure 8a). When SOD and catalase were used in combination, no additive effect was observed. When K562 cells were seeded at higher density ($6 \times 10^4$/well), they incorporated 4780 c.p.m. (± 2364, $n = 3$) and the addition of SOD and catalase resulted in slight increases in this activity (Figure 8b), but this did not reach significance ($P < 0.2$). Heat denatured enzymes (boiled for 30 min) had no effect on the rates of $^3$H-thymidine incorporation at either K562 cell density (data not shown). The addition of SOD and catalase in combination reduced the specific monocyte-dependent cytotoxicity (at a K562 seeding density of $6 \times 10^4$ cells/well) from 27% (± 4%) to 16% (± 5%) as assessed by $^{51}$Cr release ($P < 0.05$).

### Discussion

Whilst it is appreciated that monocytes possess potent cytotoxicity towards tumour cells, it is also appreciated that they do not always express such activity. Indeed, the very fact that many tumours possess large numbers of infiltrating macrophages (Dougherty & McBride, 1986) is evidence that this cytotoxic activity *in vivo* is not always expressed. Therefore, the identification of the molecular processes which regulate the development of tumouricidal competence by

**Figure 7** Effect of conditioned media from continuous cell lines on $^{51}$Cr-release from K562 cells $1 \times 10^5$ U937, HL60, HeLa or K562 cells were seeded into U-shaped wells of a microtitre plate in 200 µl culture medium and incubated for 24 h. At the end of this period the conditioned medium from each of the cell lines was removed and transferred to new U-shaped wells. To these wells, and also to control wells containing fresh culture medium, $10^4$ (in **a**) or $6 \times 10^4$ (in **b**) K562 cells loaded with $^{51}$Cr were added. Each measurement was performed in triplicate, mean values plotted and error bars show the standard deviation from the mean.

**Figure 8** Effect of SOD and catalase on $^3$H-thymidine incorporation. $10^4$ (in **a**) or $6 \times 10^4$ (in **b**) K562 cells were seeded into U-shaped wells of a microtitre plate in 200 µl of culture medium. Immediately after the addition of the cells, superoxide dismutase (SOD) (40 µg ml$^{-1}$), catalase (40 µg ml$^{-1}$) or a combination of the two were added to the wells. The cells were incubated for 24 h and 0.5 µCi $^3$H-thymidine for the last 2 h of incubation. Each measurement was performed in triplicate and the mean value plotted. Error bars show the standard deviation from the mean and controls (i.e. no catalase or SOD) were also performed in triplicate.

monocytes/macrophages offers the potential of therapeutic manipulation of these cells as a means of boosting tumouricidal protection. Murine macrophages (either resident- or elicited-peritoneal cells) are not spontaneously cytotoxic unless treated with suitable immuno-modulators such as γ-interferon and LPS (Le & Vilcek, 1984; Schultz & Kleinschmidt, 1984). However, in the present study we have shown that freshly-isolated human bloodstream monocytes exhibit considerable cytotoxicity towards cultured K562 cells in the absence of exogenously-added immuno-modulating agents. It may be therefore, that the similarities in function between murine and human monocytes/macrophages are not as closely related as is generally assumed.

In order for monocytes to exhibit maximal cytotoxicity towards the tumour cells, it appears that they must be incubated as a monolayer. This is presumably because all of the tumour cells seeded into the monocyte-containing wells then settle onto an effector cell. Hence, in these experiments it was essential that monocytes were purified to homogeneity using Nycodenz so that they could be seeded at confluence at the bottom of U shaped microtitre wells. When monocytes were isolated by combined dextran sedimentation/ficoll-paque centrifugation, an increased cell yield was obtained, but the suspensions were initially contaminated with lymphocytes: when these suspensions were cultured on plastic so that non-adherent lymphocytes were washed away, the resulting adherent monocytes were inevitably at non-confluence (Davies, 1991). Physical re-suspension of these adherent monocytes resulted in gross physical and functional damage and so this approach to purify monocytes for cytotoxicity assays was impractical. In all experiments investigating the cytotoxicity of unperturbed monocytes, it was therefore necessary to isolate cells to homogeneity by the Nycodenz method.

An important finding of the present study was the observtion that the tumour:monocyte ratio is critical in determining the outcome of the cellular interactions. Thus, at low monocyte: K562 cell ratios (3:1), cytotoxicity was observed and this was detected either as monocyte-dependent release of $^{51}$Cr or inhibition of DNA synthesis as detected by measuring $^{3}$H-thymidine incorporation. However, at higher monocyte:tumour ratios (20:1), obtained by maintaining the monocyte density but decreasing the target density, monocytes actually increased K562 cell survival (as assessed by decreasing the rate of $^{51}$Cr release) and stimulated DNA synthesis. This phenomenon was, however only observed at low seeding densities of K562 cells. The factor(s) responsible for this promotion of tumour growth and viability is endogenously-secreted from monocytes because it is present in conditioned medium and it is only effective when the target tumour cells were seeded at low cell density. It is well known that cultured cells secrete a variety of growth factors and other components which promote survival, and that these never attain sufficiently high concentrations to affect cell function when cell densities are below a critical level. Indeed, conditioned medium from several cell lines, including K562 themselves were also capable or promoting growth and survival of low density cultures. We are currently investigating whether the conditioned media from these different cell lines contains growth-promoting factors which are similar or distinct from those secreted by monocytes. It is also necessary to determine if other tumour cells, especially primary tumour cultures respond in a similar way to K562 cells. Whilst we have used two separate assays to assess tumour cell physiology, it will also be of interest to determine if other assays of cell proliferation (e.g. clonogenic assays) parallel our findings.

Levels of oxidant scavenging systems have been reported to be defective in tumour cells (Sun *et al.*, 1989) and that reactive oxidants have been implicated in tumouricidal activity either acting directly or in combination with tumour necrosis factor (Zimmerman *et al.*, 1989). Indeed, levels of catalase, glutathione peroxidase and glutathione are reduced in K562 cells compared with levels in lymphocytes (Steinkühler *et al.*, 1990). We therefore tested the effects of SOD and catalase on K562 cells. Both SOD and catalase were found to potentiate DNA synthesis in K562 cells seeded at low cell densities. This finding suggests that reactive oxidant species, possibly those generated by the tumour cells themselves, inhibit the growth and survival of these cells. it is therefore necessary to determine: (a) if the growth promoting factor(s) in conditioned media from monocytes and other cell lines is a growth factor or an antii-oxidant; (b) the role played by endogenous- and monocyte-derived reactive oxidants in tumour physiology; (c) whether monocyte-derived products play a role in enhancing tumour growth and survival *in vivo*.

We thank the North West Cancer Research Fund and the Cancer Research Campaign for financial support.

### References

CARSWELL, E.A., OLD, L.J., KASSEL, R.L., GREEN, S., FIORE, N. & WILLIAMSON, B. (1975). An endotoxin-induced serum factor that causes necrosis of tumors. *Proc. Natl Acad. Sci. USA*, **72**, 3666–3670.

CURRIE, G.A. (1981). Promotion of fibrosarcoma cell growth by products of syngeneic host macrophages. *Br. J. Cancer*, **44**, 506–513.

DAVIES, B. (1991). *An investigation of tumour cell killing by human moncotyes*. Ph.D. Thesis, University of Liverpool.

DOUGHERTY, G.D. & MCBRIDE, W.H. (1986). Accessory cell activity of murine tumor-associated macrophages. *J. Natl Can. Inst.*, **76**, 541–548.

DRATH, D.B. (1985). Enhanced superoxide release and tumoricidal activity by a post lavage *in situ* pulmonary macrophage population in response to activation. *Mycobacterium bovis* BCG exposure. *Infect. Immunol.*, **49**, 72–75.

EDWARDS, S.W., SAY, J.E. & HUGHES, V. (1987). Gamma interferon enhances the killing of *Staphylococcus aureus* by human neutrophils. *J. Gen. Microbiol.*, **134**, 37–42.

FIDLER, I.J., DARNELL, J.H. & BUDMEN, M.B. (1976). *In vitro* activation of mouse macrophages by rat lymphocyte mediators. *J. Immunol.*, **17**, 666–673.

GORELICK, E., WITROUT, R.H., COPELAND, D. & HERBERMAN, R.B. (1985). Modulation of formation of tumour metastasis by peritoneal macrophages elicited by various agents. *Cancer Immunol. Immunother.*, **19**, 35–42.

HIBBS, J.B. Jr (1974). Discrimination between neoplastic and non-neoplastic cells *in vitro* by activated macrophages. *J. Natl Cancer Inst.*, **53**, 1487–1492.

HIBBS, J.B., TAINTOR, R.R., CHAPMAN, H.A. & WEINBERG, J.B. (1977). Macrophage tumor killing: influence of the local environment. *Science*, **197**, 279–282.

HOLMES, B., PAGE, A.R. & GOOD, R.A. (1967). Studies of the metabolic activity of leukocytes from patients with a genetic abnormality of phagocytic function. *J. Clin. Invest.*, **46**, 1422–1432.

INOUE, Y. & NELSON, D.S. (1984). Effect of tumour associated macrophages on the growth of tumours in rats. *Aust. J. Exp. Biol. Med. Sci.*, **62**, 181–188.

KADHIM, S.A. & REEDS, R.C. (1984). Enhancement of tumor growth in mice: evidence for the involvement of host macrophages. *Cell. Immunol.*, **87**, 259–269.

KAPLAN, A.M. (1981). Cytostasis of tumor and non-tumor cells. In *Methods for Studying Mononuclear Phagocytes*, Adams, D.O., Edelson, P.J. & Koren, H.S. (eds). Academic Press, 775–783.

KLEBANOFF, S.J. & CLARK, R.A. (1978). *The Neutrophil: Function and Clinical Disorders*. p. 810. North-Holland, Amsterdam.

LANZA, F., FIETTA, A., SPISANI, S., CASTOLDI, G.L. & TRANIELLO, S. (1987). Does a relationship exist betwen neutrophil myeloperoxidase deficiency and the occurrence of neoplasms? *J. Clin. Lab. Immunol.*, **22**, 175–180.

LE, J. & VILCEK, J. (1984). Lymphokine-mediated activation of human monocytes: neutralisation by monoclonal antibodies to interferon-γ. *Cell. Immunol.*, **85**, 278–283.

LOVELESSE, E. & HEPPNER, G.M. (1983). Tumour associated macrophages of mouse mammary tumours. I. Differential cytotoxicity of macrophages from metastatic and non-metastatic tumours. *J. Immunol.*, **131**, 2074–2078.

MCINTOSH, J.K., MULE, J.J., KROSNICK, J.A. & ROSENBERG, S.A. (1989). Combination cytokine immunotherapy with TNFα, IL-2 and α-interferon and its synergistic antitumour effects in mice. *Cancer Res.*, **49**, 1408–1414.

NATHAN, C.F., PRENDERGAST, T.J., WIEBE, M.E., STANLEY, E.R., PLATZER, E., REMOLD, H.G., WELTE, K., RUBIN, B.Y. & MURRAY, H. (1984). Activation of human macrophages. Comparison of other cytokines with interferon-γ. *J. Exp. Med.*, 600–605.

PIESSENS, W.F., CHURCHILL, W.H. Jr & DAVID, J.R. (1975). Macrophages activated *in vitro* with lymphocytes mediators kill neoplastic but not normal cells. *J. Immunol.*, **114**, 293–299.

REED, S.G., NATHAN, C.F., PIHL, D.L., RODRICKS, P., SHANEBECK, K., CONLON, P.J. & GRABSTEIN, K.H. (1987). Recombinant granulocyte/macrophage colony-stimulating factor activates macrophages to inhibit *Trypanosoma cruzi* and release of hydrogen peroxide. *J. Exp. Med.*, **166**, 1734–1746.

RUSSELL, S.W. (1981). Quantification of cytolysis of neoplastic cells by release of chromium-51. In *Methods for Studying Mononuclear Phagocytes*, Adams, D.O., Edelson, P.J. & Koren, H.S. (eds). Academic Press, 793–799.

RUSSELL, S.W., DOE, W.F. & MCINTOSH, A.J. (1977). Functional characterisation of a stable non-cytolytic stage of macrophage activation in tumors. *J. Exp. Med.*, **146**, 1511–1532.

SCHULTZ, R.M. & KLEINSCHMIDT, W.J. (1984). Functional identity between murine γ-interferon and macrophage activating factor. *Nature*, **305**, 239–340.

STEINKÜHLER, C., MAVELLI, I., ROSSI, L., PEDERSEN, J.Z., MELINO, G., WESER, U. & ROTILIO, G. (1990). Cytotoxicity of a low molecular weight $Cu_2Zn_2$ superoxide dismutase active center analog in human erythroleukemia cells. *Biochem. Pharmacol.*, **39**, 1473–1479.

SUN, Y., OBERLEY, L.W., ELWELL, J.H. & SIERRA-RIVERA, E. (1989). Anti-oxidant enzyme activities in normal and transformed mouse liver cells. *Int. J. Cancer*, **44**, 1028–1033.

TAYLOR, M.S., STAMP, G.W.H. & BALKWILL, F.R. (1990). Investigation of cytokine gene expression in human colorectal cancer. *Cancer Res.*, **50**, 4436–4430.

YAMAMURA, Y., FISCHER, B.C., HARNAHA, J.B. & PROCTOR, J.W. (1984). Heterogeneity of murine mammary adenocarcinoma cell subpopulations. *In vitro* and *in vivo* resistance of macrophage cytotoxicity and its association with metastatic capacity. *Int. J. cancer*, **33**, 67–72.

ZIMMERMAN, R.J., ARAFINO, B.J., CHAN, A., LANDRE, P. & WINKELHAKE, J.L. (1989). The role of oxidant injury in tumor cell sensitivity to recombinant human TNF *in vivo*. *J. Immunol.*, **142**, 1405–1409.